# Case Report: A case of new-onset retroperitoneal aggressive fibromatosis after resection of solid pseudopapillary tumor of the pancreas and review of the literature

**DOI:** 10.3389/fonc.2025.1522860

**Published:** 2025-05-23

**Authors:** Yibo Wang, Hengyu Liu, Ang Li, Fang Wang

**Affiliations:** ^1^ Shandong Provincial Third Hospital, Shandong, Jinan, China; ^2^ Department of Pancreatic Surgery, General Surgery, Qilu Hospital, Cheeloo College of Medicine, Shandong University, Jinan, China

**Keywords:** aggressive fibromatosis, solid pseudopapillary tumor of pancreas, β-catenin gene mutation, surgical trauma, computed tomography

## Abstract

Aggressive fibromatosis is a rare tumor known for its intensive local invasion and postoperative recurrence. Tumors may occur incidentally or be associated with familial adenomatous polyposis. We report a case of new aggressive fibromatosis after removal of solid pseudopapillary tumor of pancreas. The patient underwent two laparotomies, the first one was radical resection, and the second was R1 resection due to invasive fibromatosis invading the surrounding blood vessels, and titanium clips were indwelled at the resection margin for further radiotherapy treatment. Both pathological diagnoses of the patients were confirmed. There have been no previous reports of a relationship between solid pseudopapillary tumor of the pancreas and aggressive fibromatosis. We believe that surgical R0 resection should continue to be sought after surgery. If there is recurrence or metastasis, radiotherapy or targeted drug therapy such as sorafenib (400 mg/day) should be performed.

## Introduction

Pancreatic solid pseudopapillary tumor (SPTP) is a rare low-grade malignancy predominantly affecting young females, with a male-to-female ratio of 1:10. Although SPTP generally exhibits indolent behavior, recurrence or metastasis occurs in less than 5% of cases. Notably, SPTP pathogenesis is strongly linked to somatic mutations in exon 3 of the CTNNB1 (catenin Beta 1) gene, leading to nuclear accumulation of β-catenin and constitutive activation of the Wnt/β-catenin signaling pathway.

Aggressive fibromatosis (AF), a monoclonal myofibroblastic proliferation with locally invasive growth but no metastatic potential, shares a similar molecular hallmark: over 85% of AF cases harbor CTNNB1 mutations or APC gene alterations, resulting in dysregulated Wnt/β-catenin signaling. While AF has been sporadically reported following surgeries for gastrointestinal malignancies (e.g., colorectal and gastric cancers), its association with SPTP remains undocumented. Current literature hypothesizes that surgical trauma and β-catenin pathway activation may synergistically drive AF development, yet direct clinicopathological evidence is lacking.

Here, we present the first case of retroperitoneal AF arising 3 years after curative resection of SPTP. Both tumors demonstrated β-catenin nuclear positivity, suggesting a shared oncogenic mechanism. This case provides critical clinical support for the hypothesized link between Wnt/β-catenin dysregulation, surgical intervention, and AF pathogenesis, while highlighting challenges in postoperative surveillance and management of rare secondary tumors.

## Case report

### First admission

**Figure 1:**
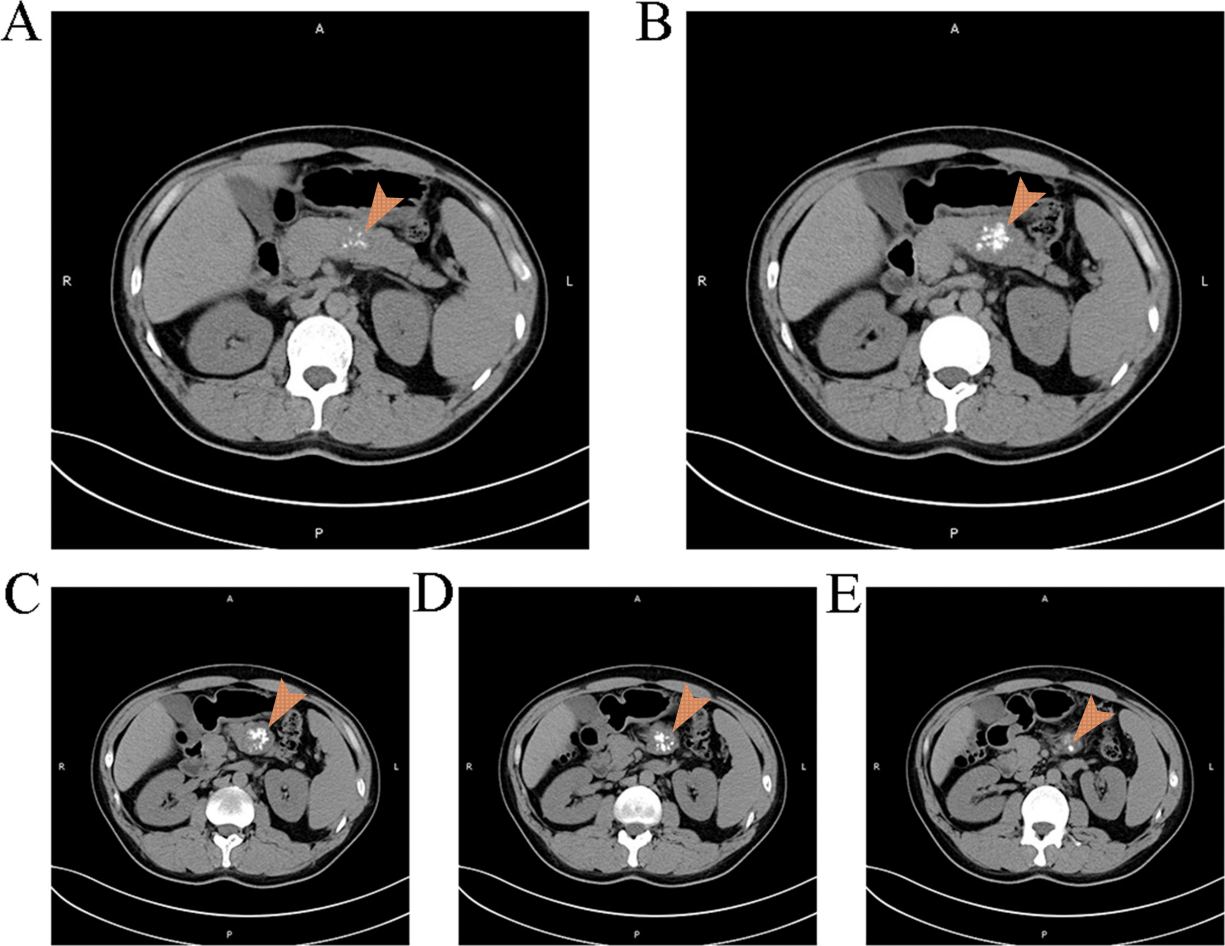
Findings of the patient's first CT scan It can be observed a space occupying lesion with clear boundaries and multiple calcifications in the pancreas.

**Figure 2:**
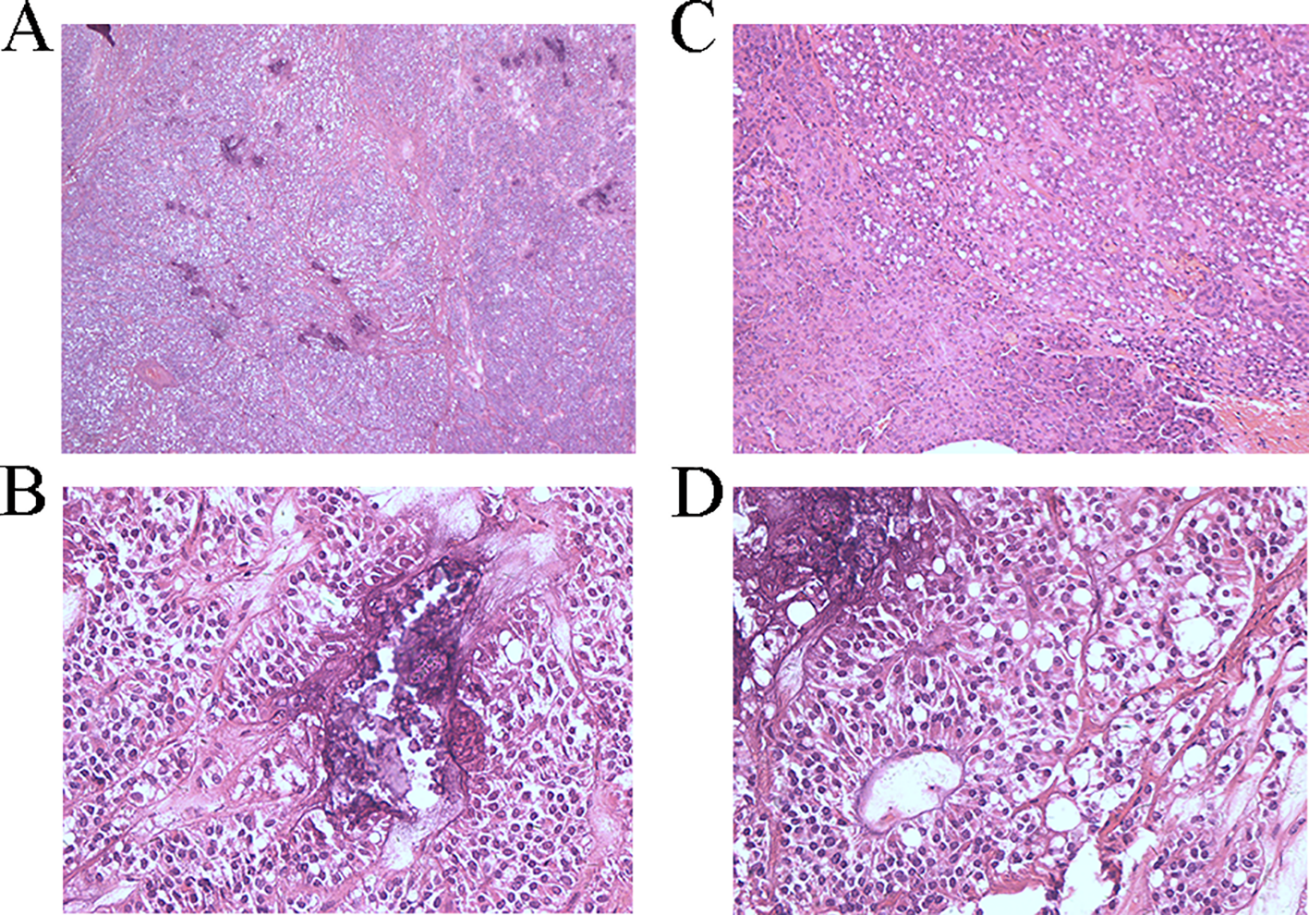
**(A)** Pathological section under 40x magnification Under the microscope, cells can be observed to be transparent or eosinophilic, with nest like growth characteristics resembling neuroendocrine tumors **(B)** Pathological section under 200x magnification Under the microscope, the boundary between the tumor and the pancreas is unclear **(C)** Pathological section under 400x magnification Under the microscope, calcification lesions can be observed **(D)** Pathological section under 400x magnification Under the microscope, nipple like structures can be observed.

A 39-year-old male was admitted due to “pancreatic occupying lesion for more than half a month” on March 20, 2021. Contrast-enhanced computed tomography (CT) of upper abdomen (March 19, 2021) showed space-occupying lesions in the body of the pancreas, with the maximum section of about 3 × 4 × 3.5 cm, clear boundary and multiple calcifications (**Figure 1**). Enhanced scan showed heterogeneous delayed enhancement of the solid part of the mass, and the fat space around the lesion was clear. The patient reported no discomfort symptoms, and specialist physical examination showed flat and soft abdomen without tenderness or rebound tenderness. The patient showed no abnormality in the relevant preoperative examination and underwent an open pancreatectomy on March 23, 2021. Intraoperative findings showed that the tumor was located in the body of the pancreas, hard in consistency, and about 3 cm in diameter, with clear borders and no surrounding tissue invasion. Then it was decided to perform spleen-preserving distal pancreatectomy, after an uneventful surgery, the specimen was removed en bloc. After the operation, this patient had few pancreatic fistula, bleeding, and gastrointestinal dysfunction, and was discharged 10 days after the operation. Postoperative pathology showed: (pancreatic body and tail) solid pseudopapillary tumor, cut area about 4 × 3.5 cm, and the boundary was not clear, the tumor was immediately adjacent to the pancreatic capsule, there is no neoplasm was found at the resection margin (**Figure 2**) Immunohistochemically: β-Catenin + (nuclei), P504S +, CD10 +, vimentin +, CD56 +, Syn foci +, CGA −, CK −, Ki-67 + (1%). (**Supplementary Figures 1-9**)

### Second admission

**Figure 3:**
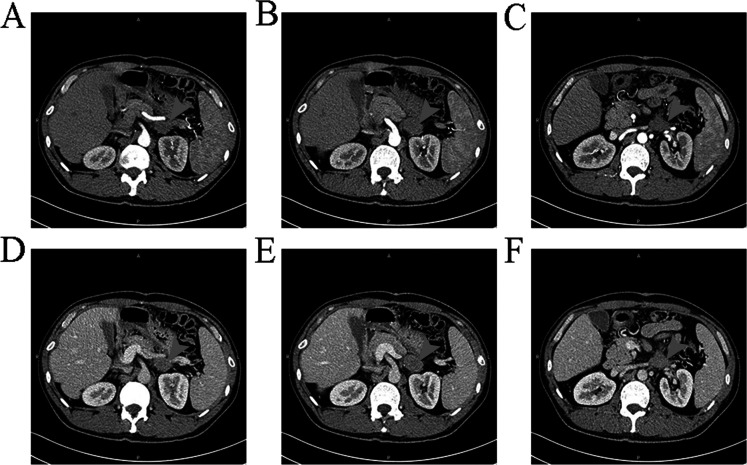
Findings of the patient's second CT scan It can be observed a space occupying lesion with moderate uniform enhancement and unclear boundaries with the left lateral adrenal branch and the outer wall of the gastric antrum.

Three years later, on April 22, 2024, the patient was re-admitted due to “retroperitoneal space occupying lesion for more than 6 days”. Contrast-enhanced CT of upper abdomen (April 17, 2024) showed a solid retroperitoneal mass in the left upper quadrant, the tumor’s section of about 2.5 × 3.5 cm, showing moderate homogeneous enhancement, the limit between the mass and the lateral branch of the left adrenal gland and the outer wall of the antrum was unclear, as well as the left renal vein was compressed (**Figure 3**). Finally, this patient was admitted as a “retroperitoneal mass”. Since its discovery, the patient had few discomfort symptoms. Physical examination showed no jaundice in the skin mucosa and sclera of the whole body, flat abdomen, and “scar hyperplasia” phenomenon in the surgical incision at the middle of the abdomen. The upper abdomen and left waist were tender without rebound tenderness, and the abdominal muscles were soft. The initial diagnosis was a retroperitoneal mass. Relevant laboratory tests: AFP: 1.70 ng/ml (0~15 ng/mL); CEA: 1.80 ng/ml (0~5 ng/mL); CA125:7.04U/ml (0-35U/ml); CA199: < 0.60U/ml (0-40U/ml). Liver function/biochemistry/blood routine/coagulation showed no abnormality.

**Figure 4:**
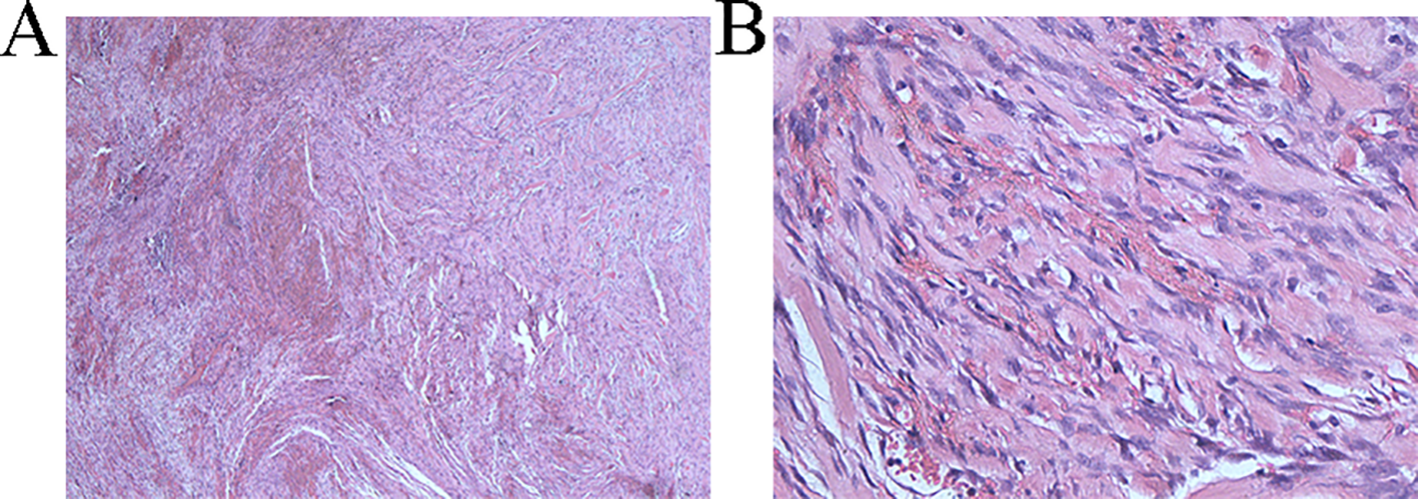
**(A)** Pathological section under 40x magnification The tumor is arranged in a bundle like pattern, with collagen bundles visible in the stroma and multiple fissures **(B)** Pathological section under 400x magnification Fibroblast cytoplasm is lightly stained or eosinophilic, with small nucleoli visible, significant interstitial bleeding, and numerous collagen fiber bundles visible.

On April 25, 2024, the patient underwent surgery. From the original incision into the abdomen, severe intra-abdominal adhesions were observed. After careful separation, the gastrocolic ligament was opened, and the vessels were cut and ligated until the splenocolic ligament, fully exposing the location of the pancreas. Exploration showed that the tumor was located behind the broken end of the pancreas and was closely related to the surrounding tissues, about 4 × 4 cm in size, hard in consistency, and with unclear borders. The upper edge of the tumor was adhered to the posterior wall of the stomach, and the lower edge was adhered to the transverse colon. After careful separation, the posterior wall of the tumor was explored, and it was seen that the tumor completely invaded the left renal vein and left renal artery. If radical resection was performed, left nephrectomy was required. Then part of the tumor tissue was taken for intraoperative rapid pathological examination, which was diagnosed as a benign tumor, which may be a spindle cell tumor. Tumor resection was continued, the tumor was carefully dissected along the anterior wall of the left renal vessel without macroscopic residual tumor, and titanium clips were placed in the area around the tumor boundary for possible later radiation therapy. Finally, the abdominal cavity was washed with distilled water and closed layer by layer. Postoperative routine pathology showed (retroperitoneal) soft tissue spindle cell proliferative lesions, involving striated muscle growth (**Figure 4**), and histomorphology combined with immunohistochemical findings, which were diagnosed as aggressive fibromatosis. Immunohistochemistry: S100 (-), SOX10 (-), vimentin (+), β-Catenin + (nucleus), Ki67 + (30%), CD68 + (histiocytes), CD34 (-), SMA + (partial), desmin (-), CD117 (-), Syn (-), CK (-), CD10 (-). (**Supplementary Figures 10-13**). We conducted follow-up up to January 2025, and no recurrence was found in the patient.

## Discussion

Solid pseudopapillary tumor of the pancreas (SPTP) is a rare low-grade malignant pancreatic tumor (1)with a significant female predisposition, a male-to-female incidence ratio of about 1∶10 (2)Some patients have few obvious clinical symptoms, and serological tests lack sensitive tumor markers. Very few SPTP will develop recurrence or metastasis (3).

The etiology and pathogenesis of SPTP are still unclear, and it is believed that the occurrence of SPTP may be closely related to β-catenin gene mutations (2, 4, 5). In recent years, it has been found that almost all SPTP have somatic mutations in exon 3 of the CTNNB1 gene encoding β-catenin protein, which leads to nuclear translocation of β-catenin, allowing β-catenin to escape cytoplasmic phosphorylation and abnormally accumulate in the nucleus, forming a β-catenin- T cell binding factor (Tcf)/lymphoid enhancer binding factor (lef) complex by binding to Tcf/lef and activating transcription factors, which further activates the transcription of multiple oncogenes (e.g., c-myc and cyclin D1) (6). Also, because β-catenin is the main regulatory molecule of the Wnt signal transduction pathway, this allows the Wnt/β-catenin signaling pathway to be activated, leading to the development of tumors (7–10)

AF, also known as desmoid tumors (DTs) and ligamentous tumors (DF), is a rare tumor characterized by monoclonal proliferation of myofibroblasts in muscles, tendons, and ligaments (11). The disease is morphologically benign but has a biological behavior of low malignancy, infiltrative growth, and a tendency to recur locally but not metastasize. In 2006, the new WHO pathology and genetics classification of soft tissue and bone tumors defines them as junctional tumors of the soft tissues, also known as mesenchymal tumors. In 2013, the World Health Organization classified them as locally invasive nonmetastatic mesenchymal cell tumor group. Its incidence is low and few clinical cases have been reported.

The pathogenesis of AF is still not fully understood, and studies have shown that activation of the Wnt/β-catenin/APC signaling pathway caused by mutations in the oncogenes APC and β-catenin (12, 13) plays an important role in the development of AF (14–18). Mutations in exon 3 of the CTNNB1 gene, which encodes a serine- and threonine-containing β-catenin sequence, are detected in 87% of AF tumors (19, 20). These mutations dephosphorylate β-catenin, which in a stable state, is not broken down by the proteasome, and can enter the nucleus and accumulate inside it, where it acts on the Tcf/lef family of transcription factors and other specific target genes such as cellular-myelocytomatosis viral oncogene (c-MYC), Matrix metalloproteinase-7 (MMP7), neural cell adhesion molecule (Nr-CAM), cyclin D1 and Cyclooxygenase-2 (COX-2) to promote cell proliferation. The accumulation of these genes in the nucleus is stimulated, which in turn activates the transcription of the target genes, thus promoting the proliferation of tumor cells. Meanwhile, elevated levels of free β-catenin in the cytoplasm play an important role in wound healing and fibroproliferative disorders and AF development (21, 22).

A few sporadic AFs and AFs associated with FAP have mutations in the APC gene, which is mutually exclusive with the CTNNB1 gene, but also acts through the Wnt/β-catenin/APC signaling pathway by inhibiting β-catenin dephosphorylation. Thus, familial adenomatous polyposis is a risk factor for aggressive fibromatosis (23–25).

And in this case report, the patient with a new onset of aggressive fibromatosis was found more than 3 years after pancreatic caudal resection for SPTP. The tumor was located in the left adrenal region - near the left renal hilum, and it occurred close to the pancreatic dissection and was considered to be of retroperitoneal origin. The author’s review of the literature revealed that there are no reports of the same in the literature. Only one similar report was found by Navin and Mafficini et al. (26, 27), describing three cases of new invasive fibromatosis after surgical resection in patients with pancreatic cancer. The common features between these articles and this case report are: all were pancreatic tumors; all were open pancreatic body tail + splenectomy (some were also combined with gallbladder and other organs resection). However, the new irregular solid soft tissue mass discovered in this case report is located near the edge of the pancreatic incision and has been confirmed by pathology to be invasive fibromatosis, while the mass discovered by Navin et al. is located in the mesentery or the epiploon retrocavity. In addition, Eric S Weiss et al. reported a case of a 63-year-old white male who underwent pylorus preserving pancreatectomy for insulinoma. After surgery, he developed aggressive fibromatosis and underwent further surgical resection, including distal pancreatectomy and total splenectomy, with only a 6cm portion of the neck and proximal body of the pancreas preserved (28). Shahab Shayesteh et al. reported a 66-year-old male with a history of colon cancer metastasis who underwent sigmoidectomy and wedge resection of the liver. Three years after surgery, a CT scan revealed new low-density lesions with unclear boundaries in the body of the pancreas. Initially, colon cancer metastasis was considered, but the puncture biopsy results ruled out this possibility. To further clarify the condition, the patient underwent distal pancreatectomy and splenectomy, and the intraoperative pathological results confirmed the diagnosis of invasive fibroadenoma of the pancreas (29).

The relationship between pancreatic adenocarcinoma and invasive fibromatosis was mentioned in this literature as unclear. Considering that the new occurrence of AF after resection of pancreatic adenocarcinoma in three cases was not entirely coincidental, researchers analyzed the similarities in pathogenesis between pancreatic cancer and invasive fibromatosis, and found that all of them were related to the specific expression of the β-catenin pathway (30, 31). Besides, the pathogenesis of pancreatic solid pseudo-papillary neoplasia is also associated with it, so this case report can provide another piece of evidence in favor of the conjecture of this literature.

In other literature reports, colorectal cancer (32, 33), gastric cancer (34, 35) gastrointestinal mesenchymal stromal tumors (36)and other malignant diseases have been reported to be complicated with invasive fibroblastoma after surgery, and although these cases did not mention whether the first tumor expressed β-catenin, the authors reviewed the relevant literature to show that β-catenin has a very high positive expression in gastric cancer (37), colorectal cancer (38)and gastrointestinal mesenchymal tumors (39)and has a certain role in the development of this tumor and the proliferation and invasion of cancer cells. The authors reviewed the relevant literature, which showed that β-catenin was highly expressed in gastric cancer (37), colorectal cancer (38), and gastrointestinal mesenchymal stromal tumor (39), and it had a certain role in the development of the tumor and the proliferation and invasion and metastasis of the cancer cells (40–42). However, neurofibromatosis may also be associated with surgery or trauma, and surgical trauma is a predisposing factor for most abdominal and extra-abdominal aggressive fibromatosis (43). The incidence of postoperative complications of invasive fibroma after laparoscopic colectomy has been reported to be significantly smaller than that of open colectomy (44), implying that less surgical stimulation may reduce the incidence of postoperative invasive fibroma. However, cases of neurofibromatosis following surgery are very rare, and given this low number compared to the number of intra-abdominal surgeries performed each day, the relatively small impact of surgical trauma, and the fact that the majority of primary tumors have specific expression of the β-catenin pathway, the Wnt/β-catenin/APC pathway can be considered a potential etiology (20, 45).

### Differential diagnosis

Intra-abdominal invasive fibromatosis is a rare tumor with irregular shape, clear margins, expansive growth, uneven density, no obvious hemorrhagic necrosis, and progressive inhomogeneous enhancement on dynamic enhancement scan. Due to the lack of clinical symptoms and imaging characteristics, it is difficult to diagnose it before surgery, especially in patients with a history of trauma or surgery at the primary site. For example, in this case, we considered the recurrence of SPTP or gastrointestinal mesenchymal tumor before surgery, and performed surgery. Intraoperatively, we found that the tumor completely encroached on the left renal artery and could not be resected by R0/R1, and we could only perform a palliative resection.

### Treatment

Therefore, for this kind of secondary surgery, patients with β-catenin positive first tumors should be fully considered for intra-abdominal invasive fibromatosis, and preoperative evaluation should be carried out first, if radical resection is feasible, then surgical treatment should be carried out, during the operation, the tumor boundaries and scar connective tissue need to be clearly confirmed, and intraoperative rapid pathological clarification of the nature of the tumor should be carried out. In case of intra-abdominal invasive fibromatosis, R0 resection should be performed as far as possible, but it is not necessary to actively expand the resection, and if it is difficult to achieve R0 resection, functional resection of organs should be preserved and postoperative radiotherapy should be added (46). A study (47)counted 426 cases of AF and found that the 5-year progression-free survival (PFS) rate for R0 resection was 62.5%, while the difference in PFS for R0 versus R1 was not statistically significant, and R2 had a significantly poorer prognosis.

Because of the characteristics of invasive growth and persistent recurrence after surgery, if the imaging examination shows that there is important blood vessel compression, it is likely that the tumor has been densely adhered to the large blood vessels, and it is impossible to perform R0/R1 resection. At this time, it is feasible to perform puncture biopsy to clarify the pathology of the tumor. If invasive fibromatosis is confirmed, the patient’s survival is expected to be long, and active waiting and observation is feasible. Some studies have shown that more than 50% of patients can maintain stable disease for a long time or even regress, of which the proportion of tumor regression is about 20% to 30% (48, 49). However, it usually takes quite a long time (median 32 months) to observe tumor regression, and if the disease progresses significantly or becomes symptomatic during this period, endocrine therapy (50)and/or NSAIDs are required (16, 51, 52). If the treatment is ineffective, patients with intra-abdominal invasive fibromatosis have rapid progression of the disease and become symptomatic, and need to have the size of their tumors reduced immediately, then radiation, chemotherapy, or targeted therapy are available. For the patients in this article, although initial positive margins are highly susceptible to short-term recurrence, AF is, after all, different from malignant tumors, and recurrence does not imply a runaway outcome (53). The patient has been followed up for 3 months with no relevant symptoms and is currently surviving well, with no significant tumor progression seen on repeat CT at the local hospital.

## Conclusion

We report a case of a SPTP that developed a new aggressive fibromatosis in its adjacent location 2 years after resection. The pathogenesis of these two tumors remains incompletely defined, and the current literature suggests that both are closely related to activation of the Wnt/β-catenin/APC signaling pathway. Here, the author ventured to speculate on the relationship between the specific expression of β-catenin pathway in intra-abdominal primary tumors and the images of surgery and the occurrence of invasive fibromatosis in the postoperative period, which could provide an interesting speculation for the clinic although there were not enough samples for further study. Meanwhile, the author put forward his opinion on the treatment of intra-abdominal type of invasive fibromatosis, which should be R0 resection as far as possible while preserving the functionality of the organ. If R0 resection is not achievable, postoperative chemotherapy can be added to achieve a similar effect (46). For patients with no obvious symptoms, with adequate notification, they can be regularly reviewed and treated with endocrine therapy and/or NSAIDs; if the tumor invades and compresses the surrounding organs/vessels, producing serious symptoms, and patients who need to have their tumor size reduced as soon as possible can choose radiotherapy, chemotherapy or targeted therapy. Considering the good prognosis of the disease, patients with radical resection and palliative treatment can achieve long-term survival.

## Data Availability

The original contributions presented in the study are included in the article/**Supplementary Material**. Further inquiries can be directed to the corresponding author.
