# Supplementary figures and images for: Case Report: A case of new-onset retroperitoneal aggressive fibromatosis after resection of solid pseudopapillary tumor of the pancreas and review of the literature
Source: Front Oncol. 2025 May 23;15:1522860. doi: 10.3389/fonc.2025.1522860 (PMC12141008; doi:10.3389/fonc.2025.1522860)

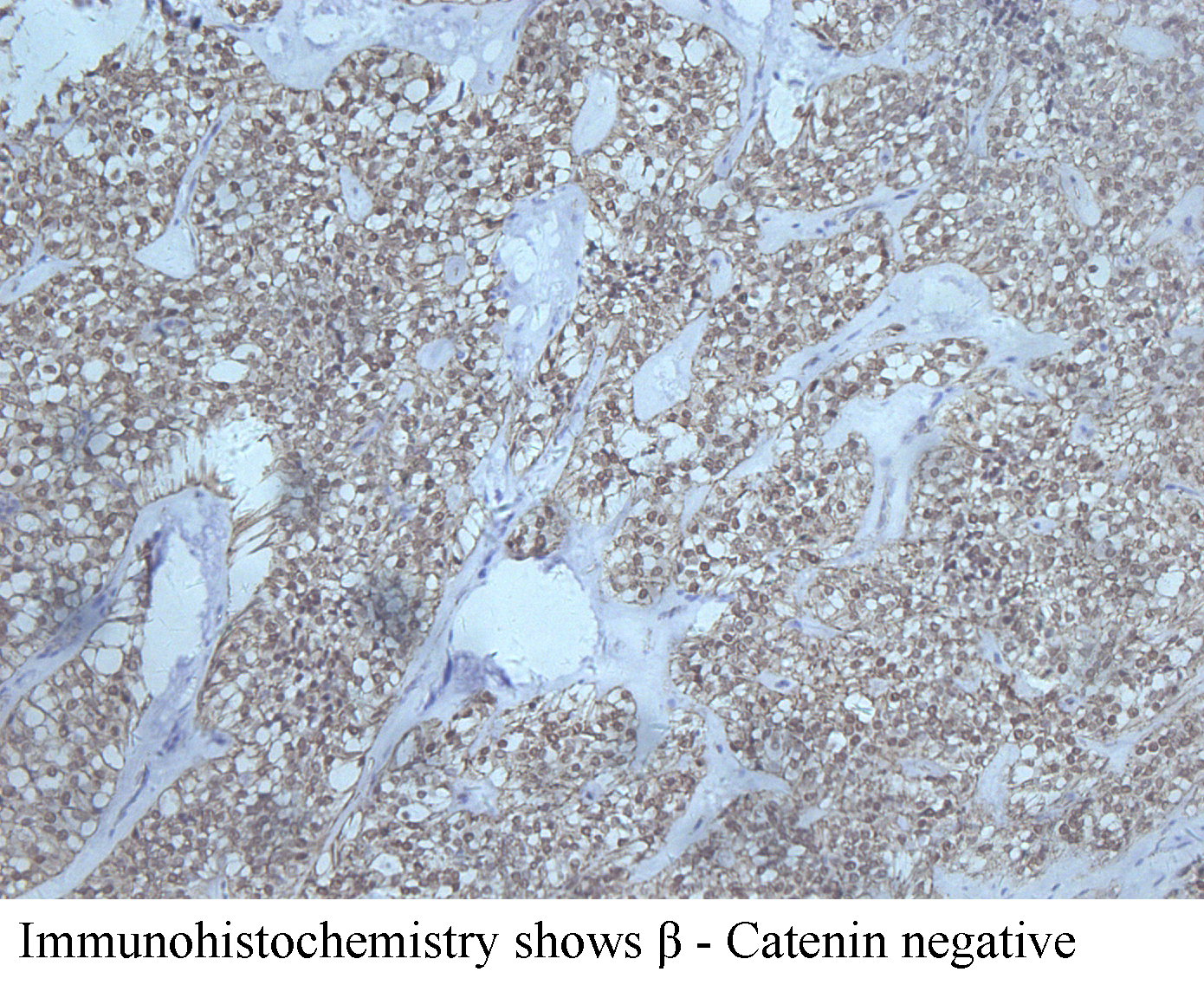

Supplement: Supplementary file 1 [file DataSheet1.zip › Supplement figure 1 β-Catenin (-).tif]

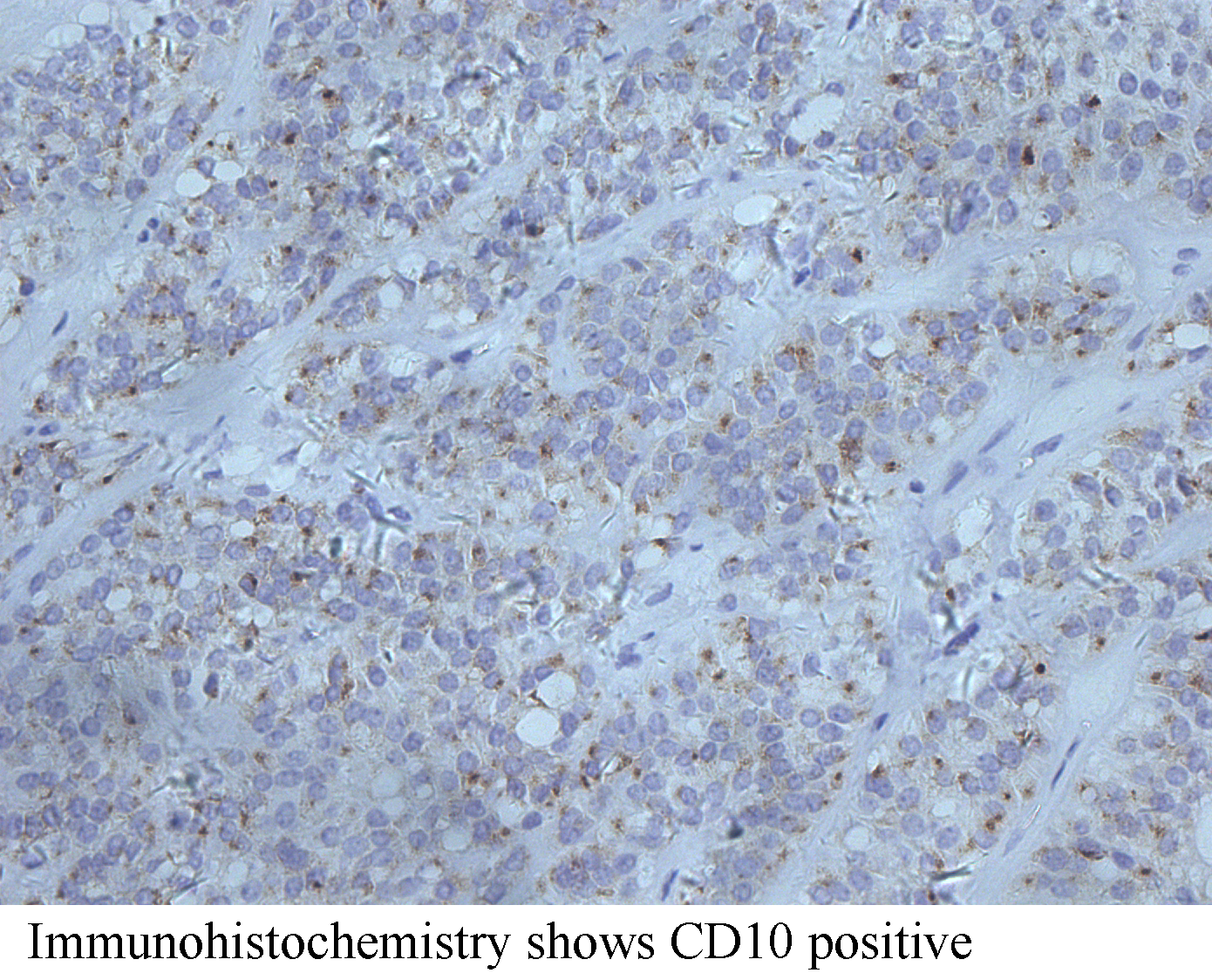

Supplement: Supplementary file 1 [file DataSheet1.zip › Supplement figure 2 CD10 (+).tif]

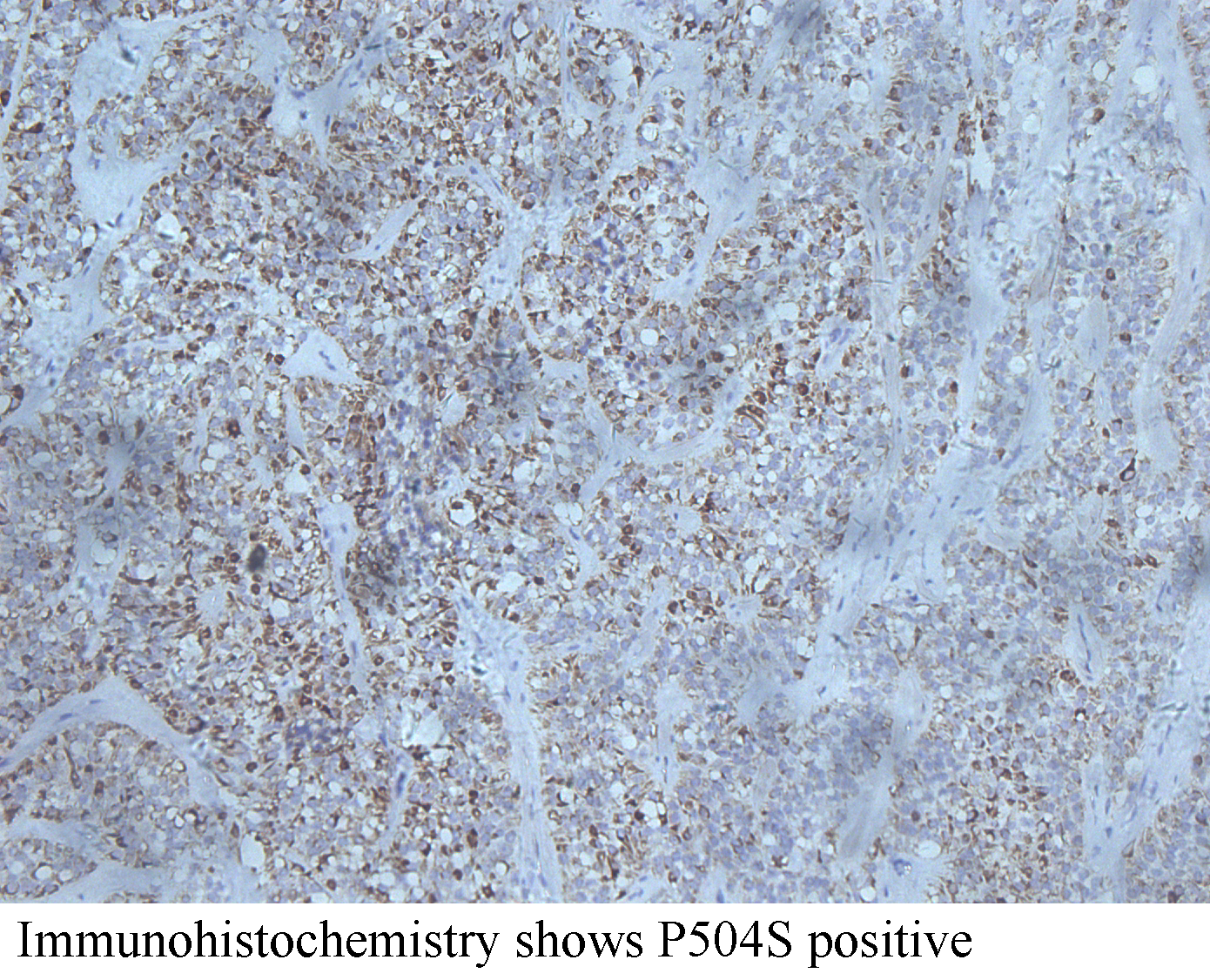

Supplement: Supplementary file 1 [file DataSheet1.zip › Supplement figure 3 P504S (+).tif]

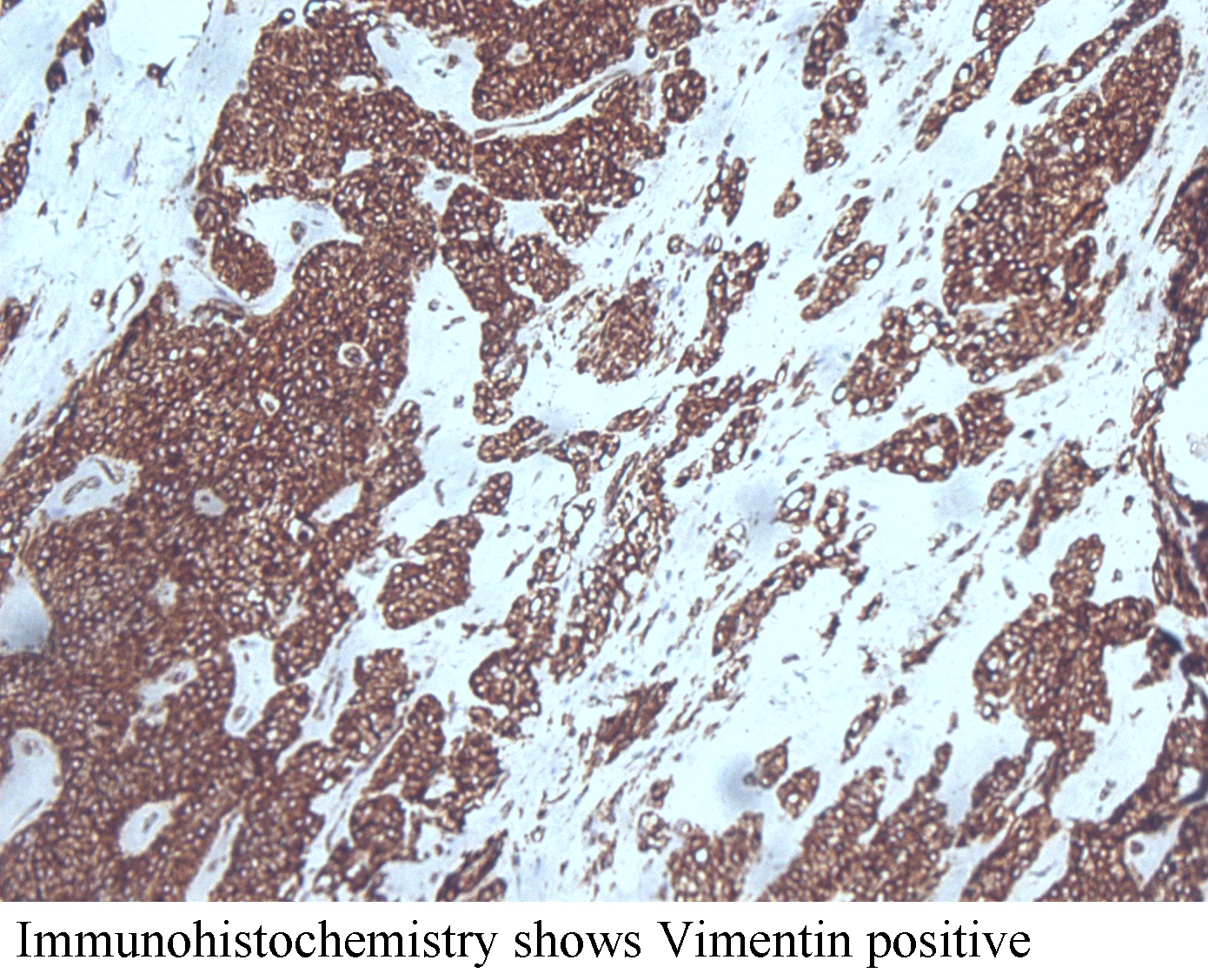

Supplement: Supplementary file 1 [file DataSheet1.zip › Supplement figure 4 Vimentin (+).tif]

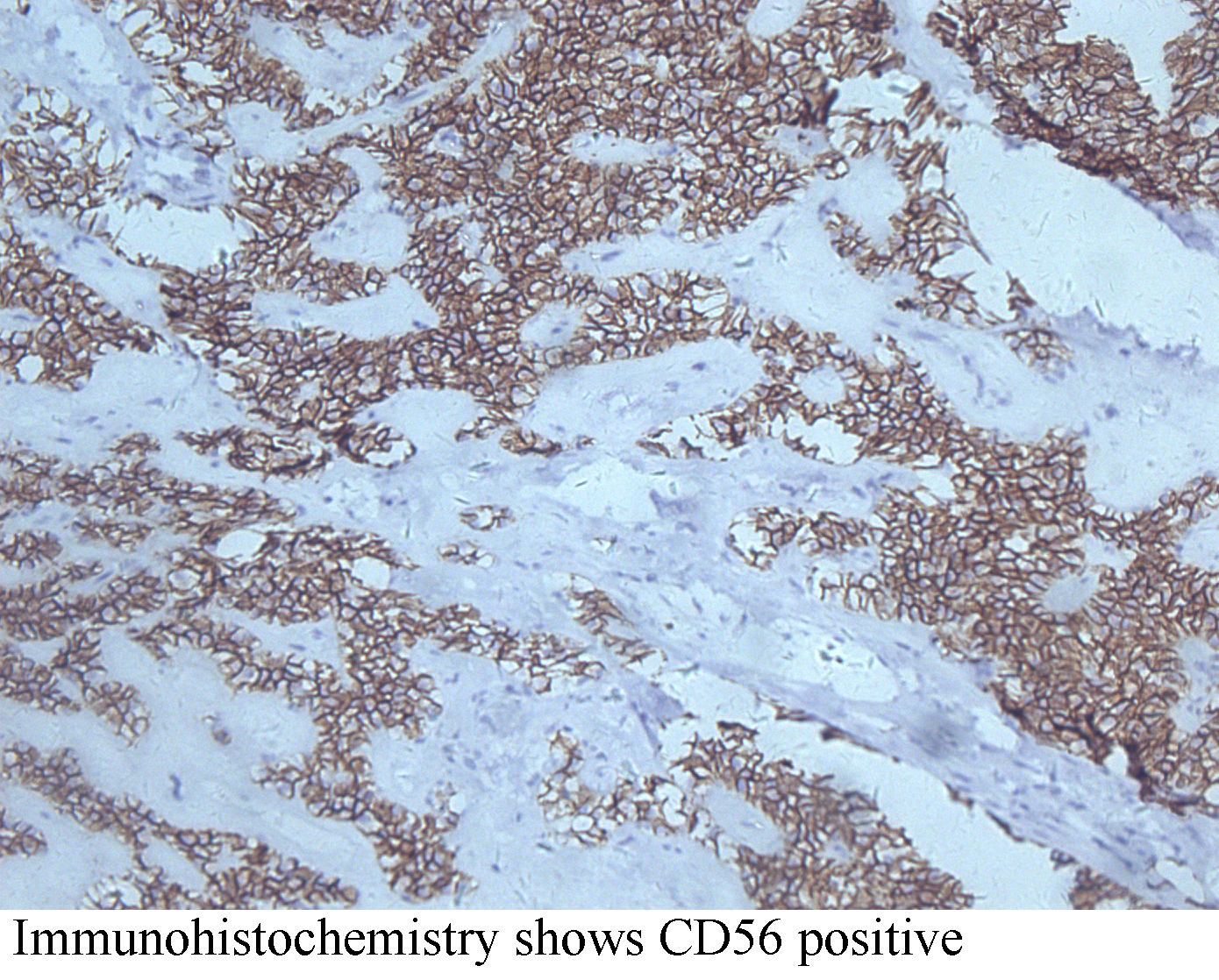

Supplement: Supplementary file 2 [file DataSheet2.zip › Supplement figure 5 CD56 (+).tif]

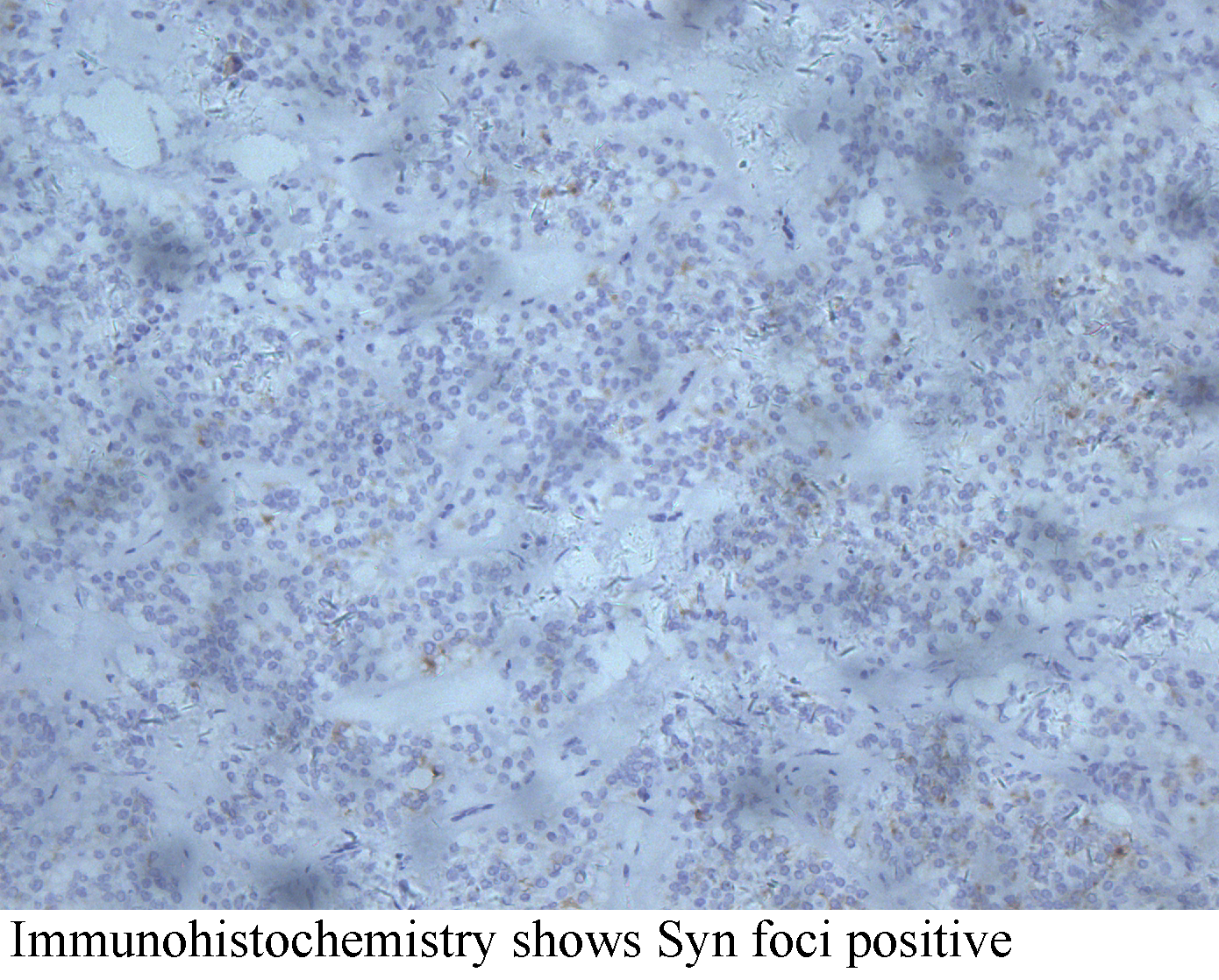

Supplement: Supplementary file 2 [file DataSheet2.zip › Supplement figure 6 Syn foci (+).tif]

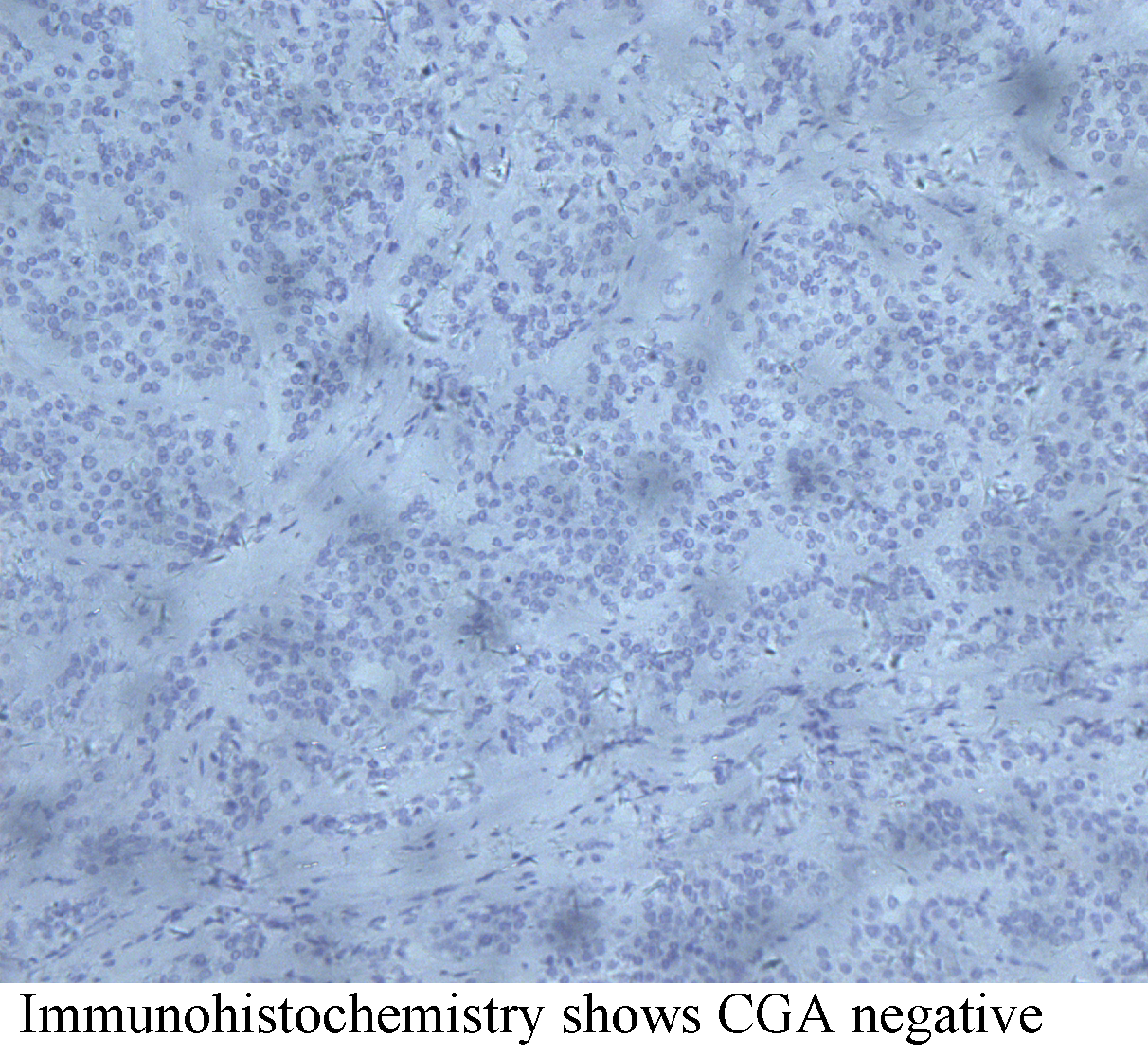

Supplement: Supplementary file 2 [file DataSheet2.zip › Supplement figure 7 CGA (-).tif]

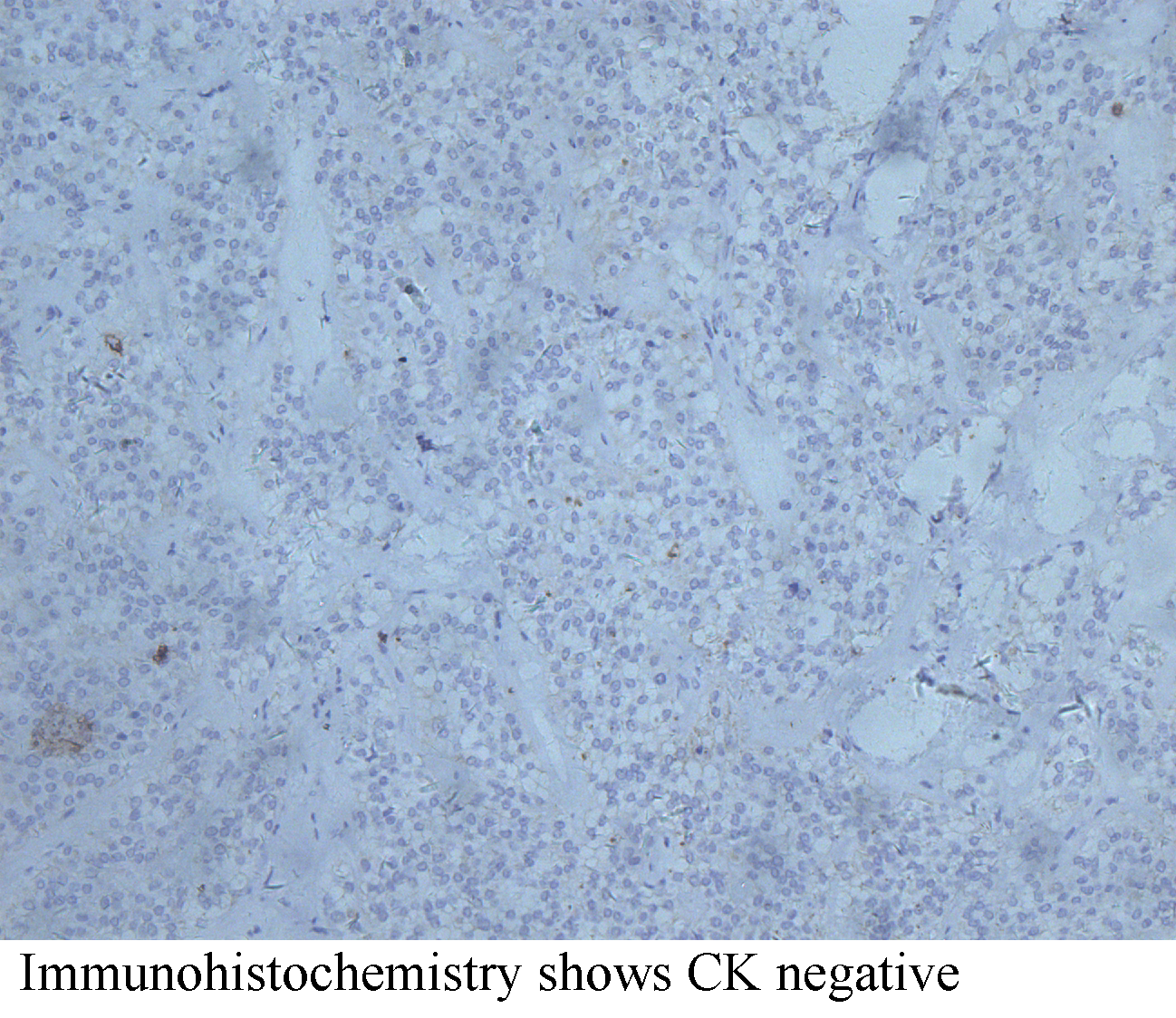

Supplement: Supplementary file 2 [file DataSheet2.zip › Supplement figure 8 CK (-).tif]

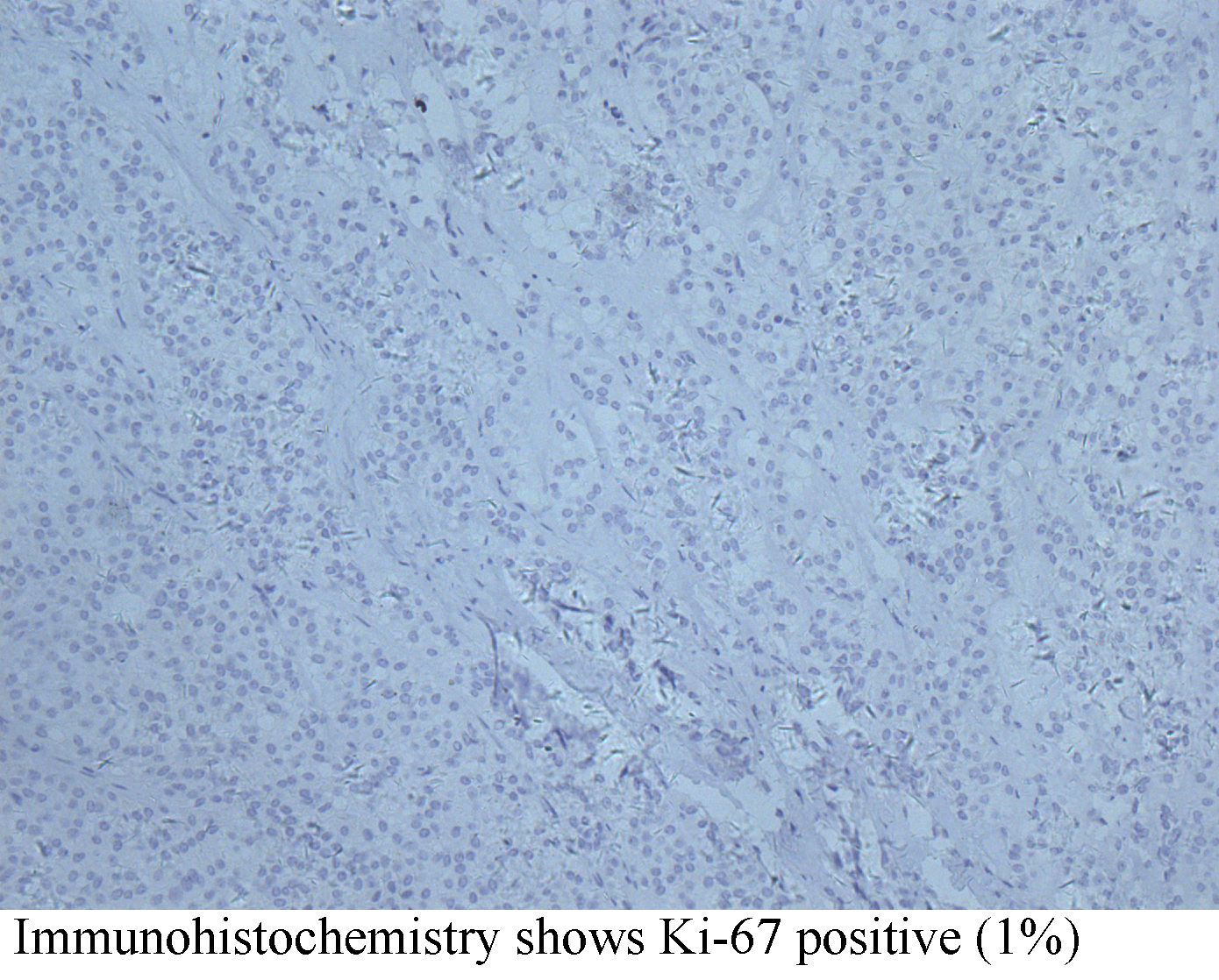

Supplement: Supplementary file 2 [file DataSheet2.zip › Supplement figure 9 Ki-67+(1%).tif]

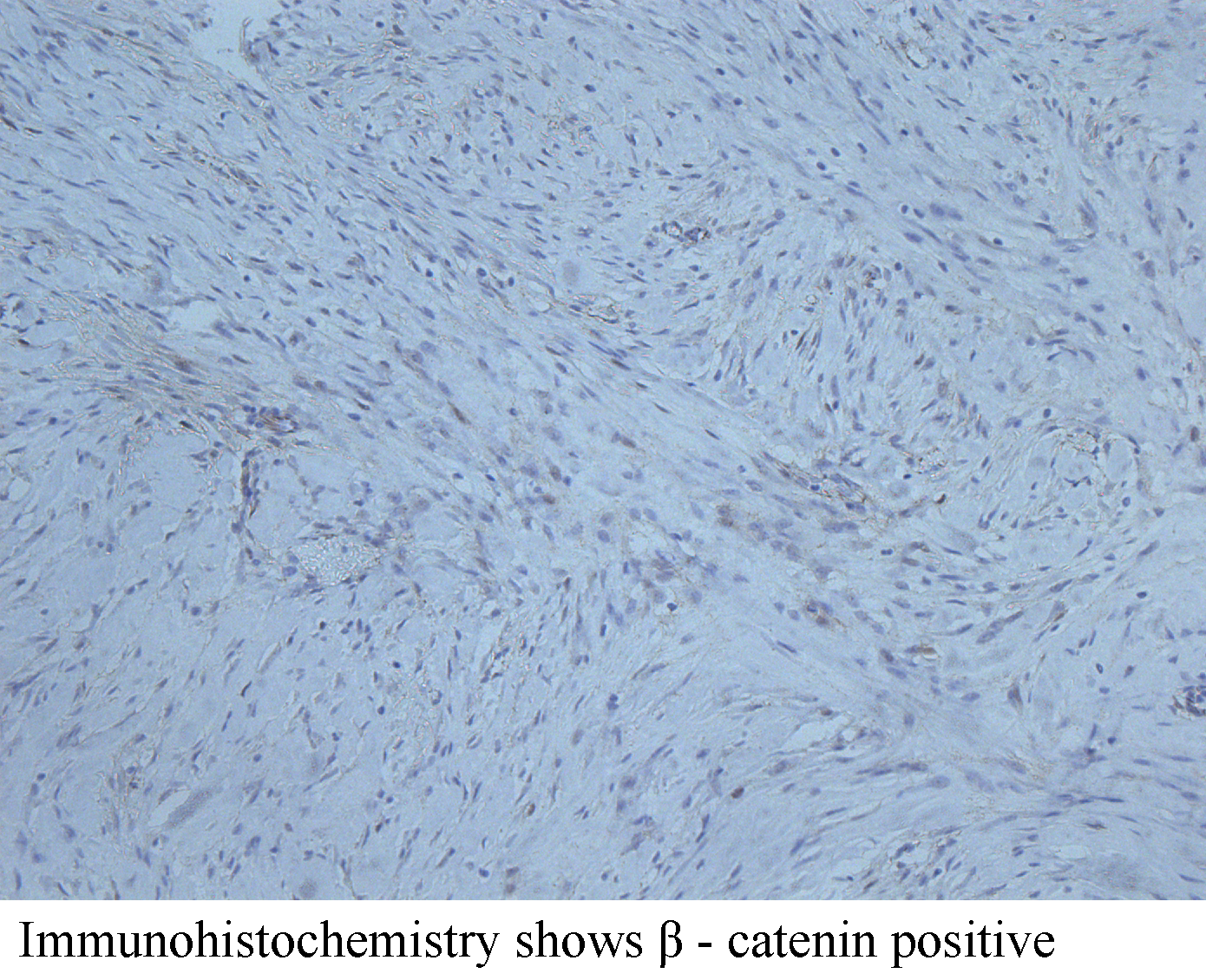

Supplement: Supplementary file 3 [file DataSheet3.zip › Supplement figure 10 β-catenin (+).tif]

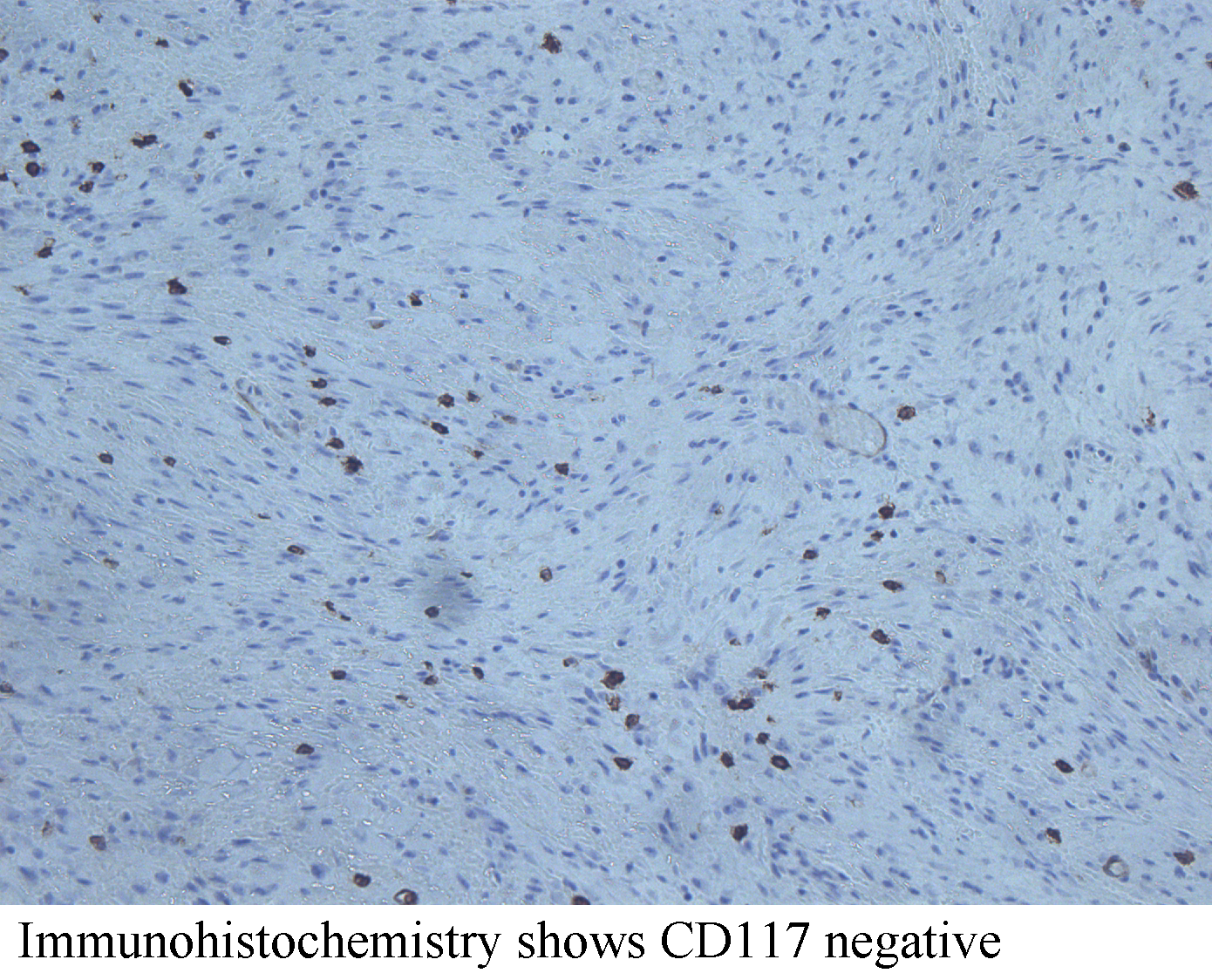

Supplement: Supplementary file 3 [file DataSheet3.zip › Supplement figure 11 CD117 (-).tif]

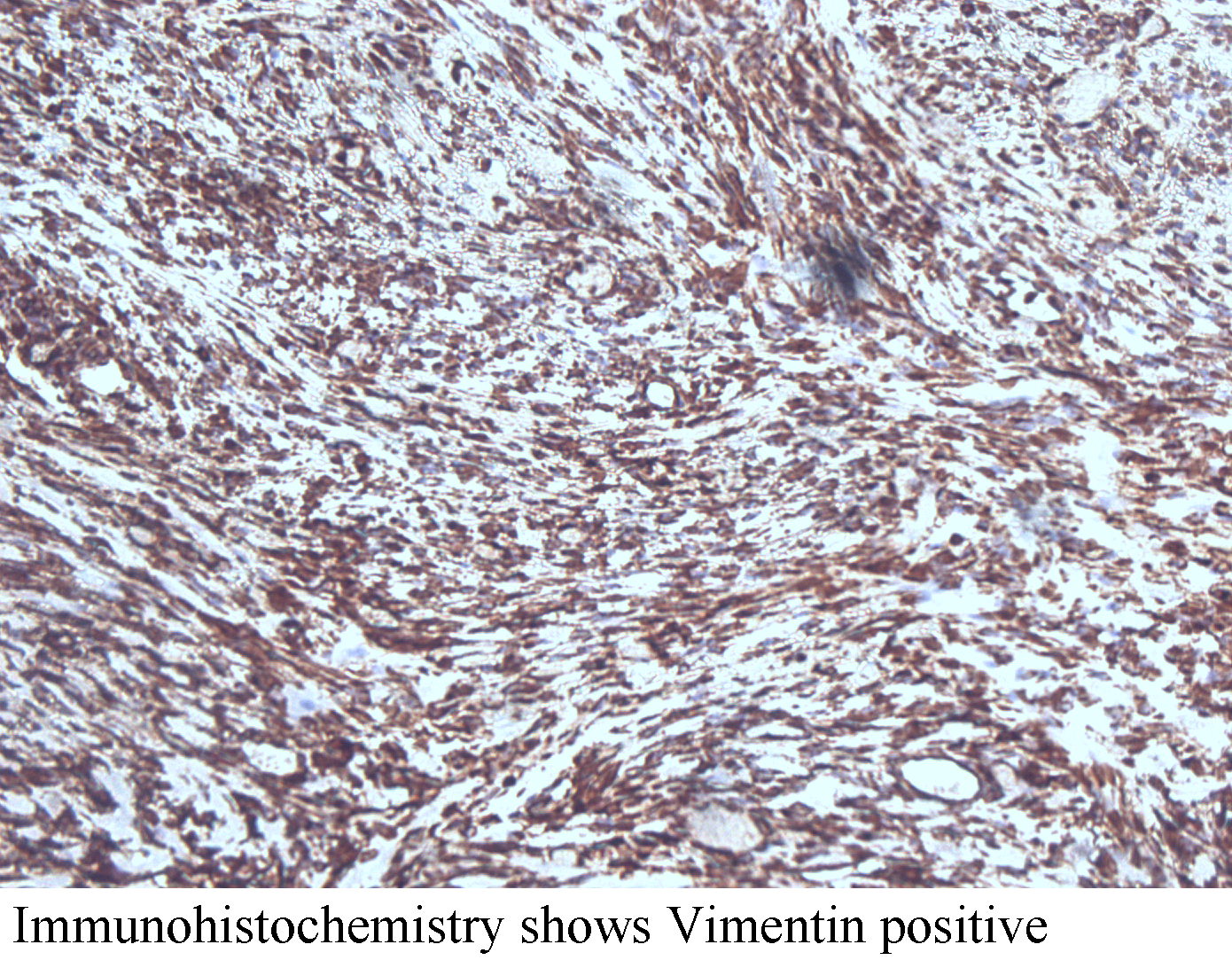

Supplement: Supplementary file 3 [file DataSheet3.zip › Supplement figure 12 Vimenti (+).tif]

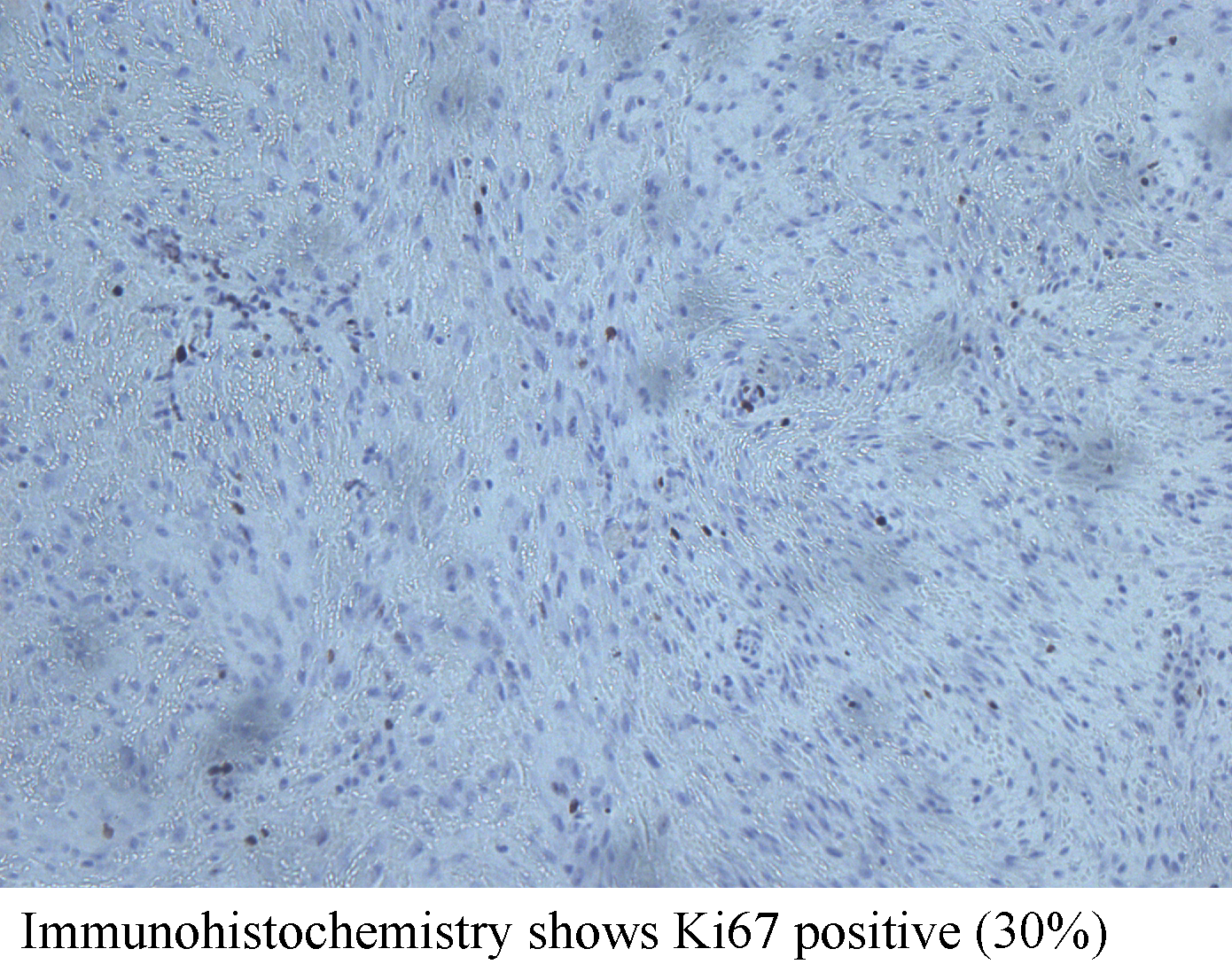

Supplement: Supplementary file 3 [file DataSheet3.zip › Supplement figure 13 Ki67+ (30%).tif]
